# Comparative of meibomian gland morphology in patients with evaporative dry eye disease versus non-dry eye disease

**DOI:** 10.1038/s41598-021-00122-y

**Published:** 2021-10-20

**Authors:** Ricaurte Ramiro Crespo-Treviño, Anna Karen Salinas-Sánchez, Francisco Amparo, Manuel Garza-Leon

**Affiliations:** 1 Department of Clinical Sciences, Division of Health Sciences, University of Monterrey, San Pedro Garza García, Nuevo León Mexico; 2 Cornea Service, Mass Eye, and Ear, Department of Ophthalmology, Harvard Medical School, Boston, MA USA

**Keywords:** Corneal diseases, Eyelid diseases, Diagnostic markers

## Abstract

Many recent studies have showed that morphological changes are one of the key signs of meibomian gland disease (MGD). These changes can be seen even before symptom onset, potentially underestimating the prevalence of MGD; however, until now, there is no conclusive information about the impact of meibomian gland (MG) morphology in tear film physiology and disease. This study aimed to investigate the prevalence of anatomical and morphological MG alterations between patients with evaporative dry eye disease (DED) and healthy controls. Retrospective chart review of seventy-five patients with evaporative DED and healthy individuals who had dry eye assessments included Ocular Surface Disease Index questionnaire, meibum quality, meibum expressibility, lid margin abnormality, ocular staining, non-invasive tear film break-up time, and meibography. We did not find significant differences in MG alterations in the upper lid between healthy and DED subjects. Patients with evaporative DED presented MG alterations in the lower lid more frequently than healthy subjects (54.8 vs. 30.3%; *p* = 0.03). The presence of shortened glands was the only MG alteration that was more prevalent in the lower lid in dry-eye patients than in healthy subjects (*p* < 0.05). Subjects with evaporative DED presented more alterations in the lower lid than healthy subjects.

## Introduction

According to the second TFOS DEWS II (the Tear Film & Ocular Surface Society Dry Eye Workshop II), dry eye disease (DED) is defined as “a multifactorial disease of the ocular surface characterized by loss of the homeostasis of the tear film, and it is accompanied by visual symptoms in which tear film instability, hyperosmolarity, damage and inflammation of the ocular surface and neuro-sensorial abnormalities play a role”^1^. DED and its symptoms are one of the most common causes of ophthalmological consultations, with a worldwide prevalence of 50–80.4%^2,3^. The most common symptoms are irritation, foreign body sensation, eye pain and redness^2^. Two types of dry eye are classically described aqueous deficient and evaporative, however, more recently patients with both components called mixed dry eye, have been described^1,4–6^.

Meibomian glands (MG) are sebaceous glands in both eyelids that provide the lipid layer of the tear film, mainly providing tear film stabilization and increasing its break-up time.

The global prevalence of MGD is 3.6–68%, and it is more common in Asian patients than in Caucasians^7^. MGD can be an asymptomatic disease that may be only detected by glandular expression and meibography, or it can be symptomatic, accompanied by signs and symptoms of DED^8^. The diagnosis in asymptomatic patients is performed based on qualitative or quantitative alterations of the MG expression^8^. There are various tools for the diagnosis of DED, such as symptom questionnaires, invasive or non-invasive tear film break- up time (NIKBUT), staining with fluorescein or lissamine green, and others^2^. Additionally, for the diagnosis of MGD lipid interferometry, slit lamp examination of anatomical changes in the eyelid, gland expressibility, quality of MG secretion, and meibography have been suggested^1,8^. Although several tools for the diagnosis and follow-up of MGD are available^7–9^, Infrared meibography is the most commonly used tool to evaluate the morphological and anatomical characteristics of MG^9,10^.

Previous studies show that morphological changes are one of the key signs of MGD^8,11^. These changes can be seen even before symptoms onset^12,13^, potentially underestimating the prevalence of MGD. For the evaluation of MGD, morphological assessment of meibography images is required, since dry eye tests cannot identify all MGD cases^14^. Although there are several studies that show a good correlation among MGD clinical parameters^15,16^, only a few have described the relationship among the MG morphologic characteristics seen in meibography and other clinical parameters, thus more studies are necessary to better understand this relationship^7,10^. Until now, there is no conclusive information about the impact of MG morphology on tear film physiology and DED. This study investigates the prevalence of anatomical and morphological alterations in MG in healthy individuals and patients with evaporative DED, and whether there is a correlation between these alterations and clinical parameters of MGD.

## Results

Seventy-five eyes of 75 subjects were studied with an average age of 40.68 ± 18.43 (range 28–78 years old), 42 of them (56%) were female, the right eye (OD) was studied in 41 (54.7%) individuals, 42 (56%) of the studied cases had dry eyes and 33 (44%) were healthy individuals. The demographic characteristics are presented in Table 1.

**Table 1:** Demographic and general characteristics.

Variable	Total n = 75	Non-dry eye n = 33	Dry eye N = 42	*p*
Right eye (%)	41 (54.7)	19 (57.6)	22 (52.4)	0.415
Female sex (%)	42 (56)	14 (42.4)	28 (66.7)	0.060
Age (years)	40.68 ± 18.43	33.70 ± 14.72	47.93 ± 20	0.007
OSDI	31.20 ± 20.12	9.18 ± 6.90	49.55 ± 20.13	< 0.001
Initial NIKBUT seconds	10.11 ± 4.30	13.86 ± 3.27	7.01 ± 1.91	< 0.001
Average NIKBUT seconds	11.86 ± 3.72	14.65 ± 2.67	9.62 ± 2.82	< 0.001
Gland expressibility^a^	1.49 ± 0.79	1.42 ± 0.70	1.55 ± 0.86	0.498
Meibium quality^b^	1.08 ± 0.85	0.97 ± 0.77	1.17 ± 0.90	0.313
MGYLS^c^	6.27 ± 2.02	9.44 ± 1.98	3.51 ± 1.69	0.004
UL meibomian drop out (%)	20.08 ± 8.04	19.29 ± 7.36	21.48 ± 8.63	0.493
LL meibomian drop out (%)	21.41 ± 7.99	21.71 ± 7.95	21.99 ± 8.043	0.761
UL Morphological alterations	1.56 ± 0.94	1.48 ± 0.87	1.66 ± 1.00	0.412
LL Morphological alterations	0.56 ± 0.70	0.36 ± 0.60	0.71 ± 0.74	0.037

non-invasive tear film break- up time (NIKBUT), Ocular Surface Disease Index questionnaire (OSDI), Upper lid (UL), Lower lid (LL).

^a^The classification used to evaluate gland expressibility was 0 = all glands expressible, 1 = 3–4 glands expressible, 2 = 1–2 glands expressible, 3 = no glands expressible according with Daniel et al. DREAM study^10^.

^b^The classification proposed by Bron et al.^33^ was used to evaluate the meibum quality as follows: clear (0), opaque (1), opaque with detritus (2), and toothpaste (3). Only the highest grade found among the expressed glands was recorded.

UL = Upper eyelid, LL = Lower eyelid.

^c^MGYLS = meibomian glands yielding liquid secretion score of the whole lid was determined according to Korb et al.^32^.

Meibomian gland morphological alterations in the upper lid (UL) were present in 68 (90.7%) of the studied subjects, while only 33 (44%) had some type of alteration in the lower lid (LL). We did not find significant differences in the presence of any given anatomical alteration in the UL between healthy and DED (92.9% vs. 87.9%; *p* = 0.69). In the LL, patients with DED presented at least one morphological alterations more frequently than healthy subjects (54.8% vs. 30.3%; *p* = 0.03). However, for any given individual alteration in the LL, only the presence of shortened glands was more common in subjects with DED than in healthy subjects. The distribution of all MG morphological alterations is presented in Table 2.

**Table 2:** Morphological alterations of meibomian glands.

Morphological alteration*	Total n = 75	Non-dry eyes n = 33	Dry eye n = 42	P*
**Upper eyelid**				
Distorted n (%)	12 (16)	3 (9.1)	9 (21.4)	0.209
Tortuous n (%)	23 (30.7)	13 (39.4)	10 (23.8)	0.207
Hooked n (%)	17 (22.7)	8 (24.2)	9 (21.4)	0.788
Drop out n (%)	1 (1.3)	0 (0)	1 (2.4)	1.00
Shortened n (%)	23 (30.7)	7 (21.2)	16 (38.1)	0.137
Thickened n (%)	1 (1.3)	1 (3)	0 (0)	0.440
Thinned n (%)	6 (8)	3 (9.1)	3 (7.1)	1.00
Overlaping n (%)	9 (12)	2 (6.1)	7 (16.7)	0.283
Ghost n (%)	13 (17.3)	3 (9.1)	10 (23.8)	0.128
Tadpoling n (%)	1 (1.3)	0 (0)	1 (2.4)	1.000
Abnormal gap n (%)	6 (8.0)	4 (12.1)	2 (4.8)	0.395
Fluffy areas n (%)	5 (6.7)	4 (12.1)	1 (2.4)	0.163
No extensión to lid margin n (%)	2 (2.7)	1(3.0)	1 (2.4)	1.000
Any alteration n (%)	68 (90.7)	29 (87.9)	39 (92.9)	0.692
**Lower eyelid**				
Distorted n (%)	5 (6.7)	2 (6.1)	3 (7.1)	1.000
Tortuous n (%)	0 (0)	0 (0)	0 (0)	
Hooked n (%)	3 (4)	0	3 (7.1)	0.251
Drop out n (%)	6 (8)	3 (9.1)	3 (7.1)	1.000
Shortened n (%)	15 (20)	3 (9.1)	12 (28.6)	0.04
Thickened n (%)	1 (1.3)	0 (0)	1 (2.4)	1.000
Thinned n (%)	0 (0)	0 (0)	0 (0)	
Overlaping n (%)	0 (0)	0 (0)	0 (0)	
Ghost n (%)	1 (1.3)	1 (3)	0 (0)	0.440
Tadpoling n (%)	0 (0)	0 (0)	0 (0)	
Abnormal gap n (%)	1 (1.3)	0 (0)	1 (2.4)	1.000
Fluffy areas n (%)	6 (8)	1 (3.0)	5 (11.9)	0.220
No extensión to lid margin n (%)	4 (5.3)	2 (6.1)	2 (4.8)	1.000
Any alteration n (%)	33 (44)	10 (30.3)	23 (54.8)	0.03

*Morphological alteration as defined in Fig. 2A–D.

The analysis by gender did not show any significant differences in the presence of MG alterations. When analyzing the prevalence of alterations by age, ghost glands in the UL were more common in subjects older than 40 years (11 vs 2, *p* 0.012, OR 7.13 IC 95% 1.45 to 34.89). Fluffy areas were more common in subjects younger that 40 years old (0 vs 5, *p* = 0.025, OR 1.15 IC 95% 1.01 to 1.31). There were no significant differences in the morphological alterations in the LL between the two age groups.

**Figure 1:**
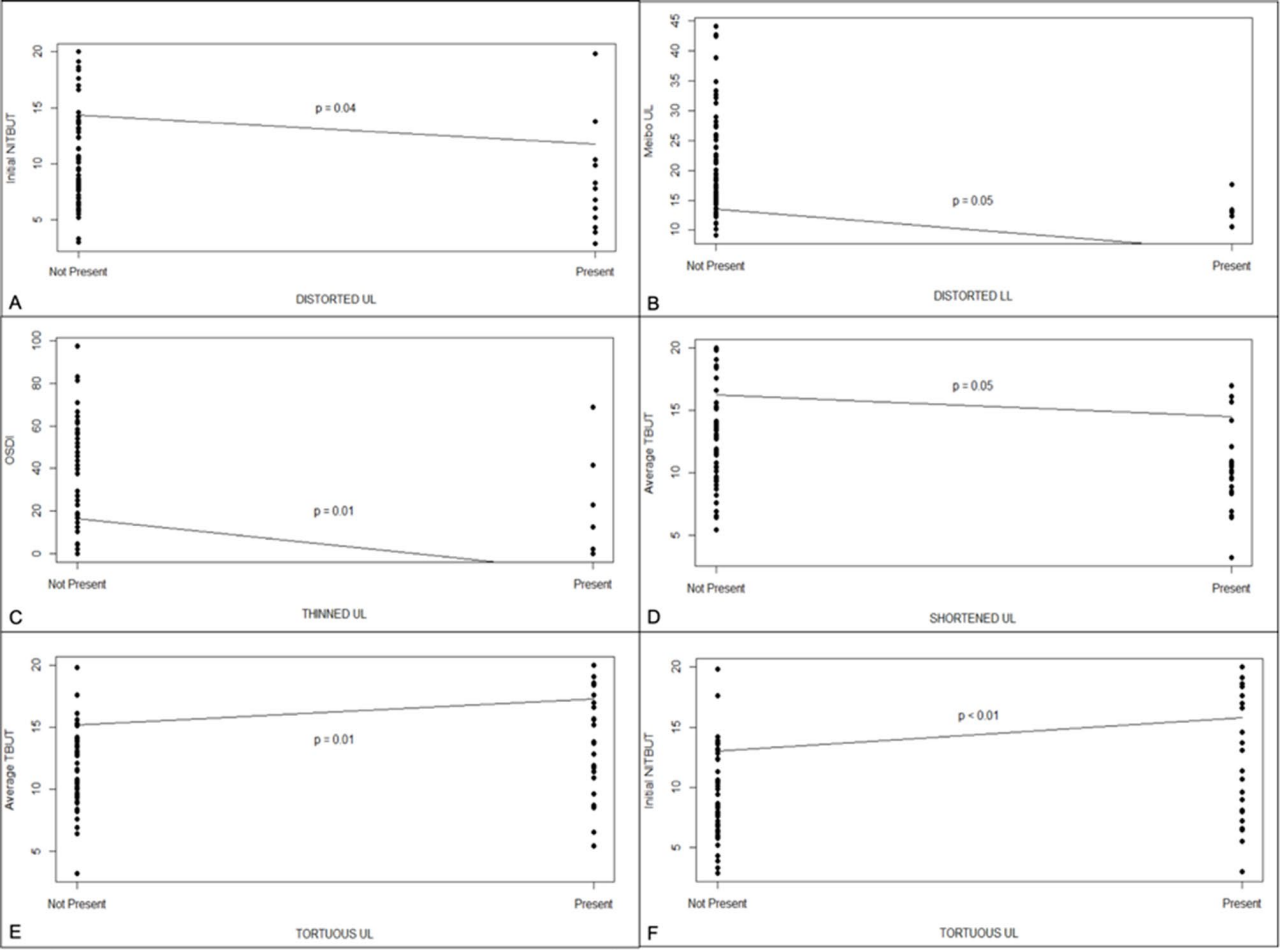
Logistic regression between clinical parameters and morphological characteristic (**A**) initial NITBUT (TBUT_I) on distorted glands in upper eyelids (UL). (**B**) Logistic regression of meiboscore on distorted glands lower eyelids (LL). (**C**) Logistic regression of OSDI on thinned glands in UL. (**D**) Logistic regression of average TBUT (TBUT_A) on shortened glands in UL. (**E**) Logistic regression of average TBUT (TBUT_A) on tortuous glands in UL. (**F**) Logistic regression of initial NITBUT (TBUT_I) on tortuous glands in UL.

The logistic regression of clinical parameters and morphological characteristics showed a statistically significant relationship between the OSDI score and thinned gland (estimate: − 25.04, *p* value: 0.01), initial NIKBUT and tortuous and distorted glands (estimate: 2.82, *p* value: < 0.01, estimate: − 2.56, *p* value: 0.04, respectively), average NITBUT and shortened and tortuous glands (estimate: − 1.73, *p* value: 0.05, estimate: 2.10, *p* value: 0.01, respectively), in UL. In the LL, there was a significant relationship between MG loss and distorted glands (Fig. 1A–F). All the other parameters did not show significant correlations.

## Discussion

The results of this study confirm the high prevalence of meibomian gland morphological alterations in patients with DED and in healthy adults. Nevertheless, MG morphological alterations were more common in the lower eyelid of patients with DED than in healthy individuals. Most of the research studies has focus on presence and severity of dropout of MG, as a marker for severity^8,11,17–19^. Recently, Daniel et al.^10^ proposed a classification system that not only evaluates the percentage of MG dropout but also considers MG morphological alterations.

Although in our study morphological alterations were more common in the UL than LL, adding to the evidence reported by Daniels et al.^10^, we also found that alterations in the LL were twice as common as those in their report. A potential explanation is that we studied only patients with evaporative DED, while Daniels et al. studied patients with all types of DED, including almost 40% with autoimmune diseases, and these patients tend to present fewer MG alterations than other types of DED^20^.

We did not find differences in morphological alterations between the two studies groups in the UL; we speculate that the significant increase in LL MG changes we observed in subjects with DED (54.8 vs 30.3%, *p* = 0.03, OR 2.78, 95% CI 1.06, 7.26), could be because of the greater influence that damage of MG in the lower eyelid has on MGD development^21,22^.

The only morphological alteration that was significantly more common in patients with DED than in healthy subjects was shortening of MG. This may be explained by the evidence showing that MG expressibility depends on residual gland length in the LL, with shorter glands been more difficult to express^23^. Recently, Singh et al. demonstrated that only short glands show atrophic changes with loss of meibocyte differentiation and cellular proliferation, while hooked, tortuous and overlapping glands have completely normal glandular histology^24^.

Interestingly, we found a statistically significant (positive) correlation between the presence of tortuous glands in the UL and longer initial and average NIKBUT. Daniel et al.^20^ reported similar results in 394 ULs, where the presence of tortuous glands was associated with a longer TBUT and Schirmer test.

Additional MG morphological alterations have been reported in patients with MGD, such as gland distortion^25^, tortuosity^26^, thinning, thickening, or with abnormal gaps^11,27^. We did not find a higher frequency of other morphological alterations in eyelids of patients with evaporative DED when compared with healthy subjects, probably because of the protocol that we followed (central-focused)^10,20^. However, other investigators have reported greater loss of MG in the nasal and temporal zones in patients with MGD^18^.

Our study has some limitations, including an older age in the DED group than in the healthy subjects group, the MG evaluation protocol that only included the central glands, and a significant lack of previous studies that limited the availability of data and prevented an adequate sample size calculation.

In conclusion, our data shows that patients with evaporative dry eye disease presented more morphological alterations in the lower eyelid than healthy subjects and the shortening of meibomian glands is a key morphological finding in patients with evaporative dry eye disease.

## Materials and methods

A retrospective chart review of patients with evaporative DED and healthy individuals was performed. The study was approved by the Research Committee and the Bioethics Committee of the Health Sciences Division of the University of Monterrey, and adhered to the tenets of the Declaration of Helsinki. Requirement for a written informed consent was waived by the Comité de Investigación de la Vicerrectoría de Ciencias de la Salud de la Universidad de Monterrey (ref: 05132020-a-OFT-CC-CI) because of the retrospective observational nature of the study and information that allows the identification of the patient was not used.

### Study population

For healthy subjects, the inclusion criteria included: an Ocular Surface Disease Index (OSDI) of < 12 points, non-invasive tear film break- up time (NIKBUT) > 10 s, and negative ocular surface staining in order to exclude any patient with DED. Patients with evaporative DED were included according to the criteria of the International Workshop on meibomian gland dysfunction, briefly, OSDI score > 12 points, NIBUT < 10 s, expressibility grades 1 to 3, and MG yielding liquid secretion (MGYLS) score < 4^8^. The exclusion criteria for both groups included any uncontrolled systemic conditions, history of refractive or eyelid surgery, corneal infection, active ocular diseases (except DED for the corresponding group), history of facial paralysis, and use of contact lenses in the previous 7 days.

### Evaluation of signs of dry eye

All assessments of signs were performed by one ophthalmologist specialist in cornea and ocular surface diseases (MGL). The following information was studied: self-administered OSDI questionnaire (Allergan, Irvine, CA) which had been validated in the Spanish language^28^, for ocular surface staining 5 μL of 2% fluorescein diluted in saline solution was instilled in the cul-de-sac, and 2 min later, corneal damage was assessed under the slit lamp using a cobalt blue and yellow filter^29^. Stratification was performed using the classification of the Ocular Staining Score^30^.

Evaluation of non-invasive tear film breakup time (NIKBUT) was calculated with the Antares topographer (Construzionne Strumenti Oftalmici, Florence, Italy) according to the manufacturer’s instructions. Briefly, two readings are provided at the end of every assessment: NIBUT-Initial, the time taken for the first appearance of a break in the tear film, and the NIBUT-Average is the average of the time taken to break-up in all the regions monitored over the 17 s. For the statistical analysis, the average of three consecutive measurements was used. To reduce the influence of the clinical tests on the results, the non-invasive studies were carried out first: questionnaire, NIKBUT and meibography, slit-lamp evaluation of the eyelid characteristics, expression of the MG, and ocular surface staining, in this order.

### Meibomian gland characteristics

**Figure 2:**
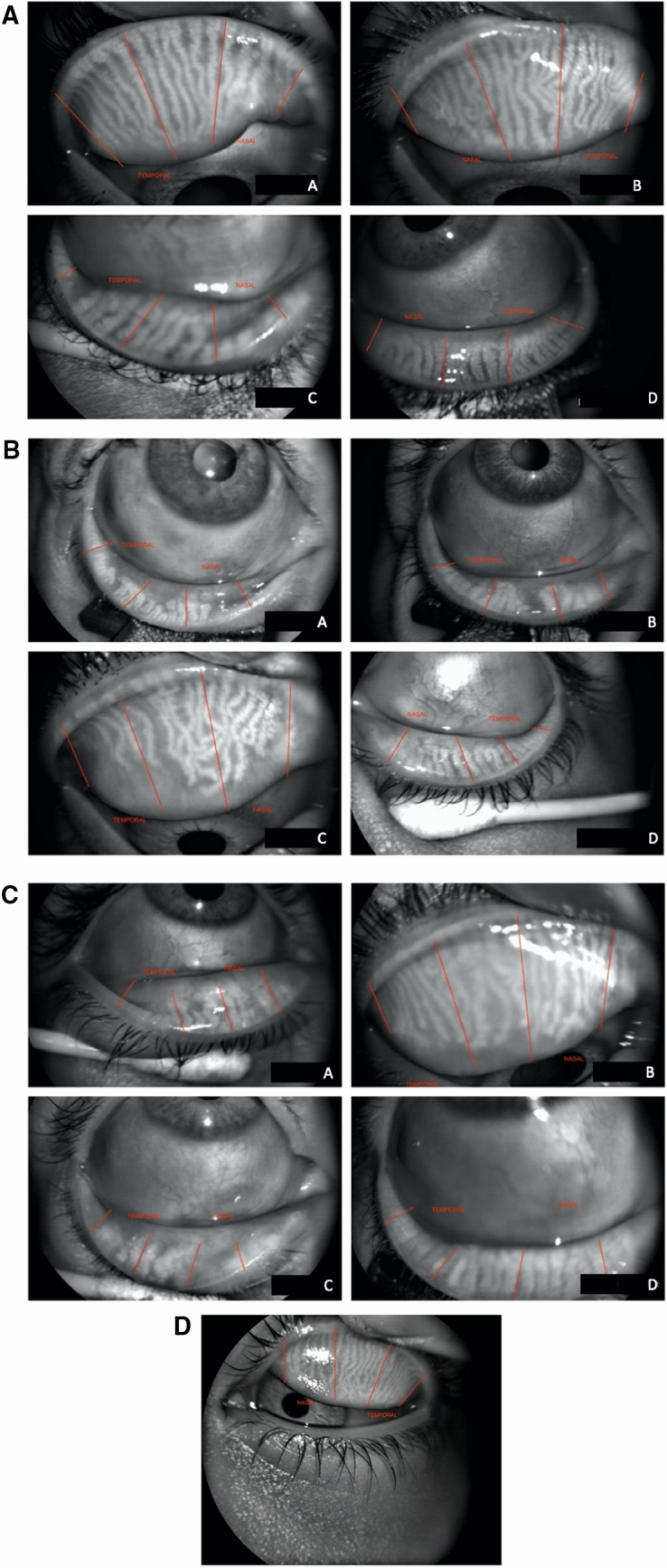
(**A**) Morphological characteristics of Meibomian Glands. (A) distorted (Distorted gland do not follow the parallel course of normal glands The distortion is less than that of a tortuous gland). (B) tortuous. (Tortuous gland must have at least one prominent tortuous configuration in the gland) (C) thinned. (Thinned/Attenuated glands have a width that is less than half the width of a normal gland) (D) thickened. (Thickened glands have a width that is equal to or more than twice the width of a normal gland). (**B**) Morphological characteristics of Meibomian Glands. (A) shortened. (The gland does not extend to its all normal length) (B) Drop out. (An empty space where a gland should have been observed. This would form part of the total dropout area along with areas from shortened glands, if such gland is present. (C) ghost. (Pale glands with absence of the normal meibomian gland architecture) (D) hooked. (Hooked glands curl back at the distal end, resembling a fishhook. (**C**) Morphological characteristics of Meibomian Glands. (A) tadpoling (Glands are thick at the eyelid margin but taper and thin out distally). (B) abnormal gap. (Gaps are normal spaces between glands. Abnormal gaps are present when two adjoining glands are pushed aside such that the width of the gap between them is at least twice that of a normal gland (C) fluffy areas (Amorphous white substance in areas where normal glands should have been present. Individual glands with sharp borders or normal architecture cannot be visualized) (D) no extension to lid margin (Gland stops short of the lid margin). (**D**) Morphological characteristics of Meibomian Glands. (A) overlapping (One gland crossing over adjoining glands. Can be under or over.

Evaluation of the MG characteristics of the upper and lower eyelids (UL and LL, respectively) included secretion, number of expressible glands, anatomical changes assessed by dropout rate, and morphological characteristics of the eyelid central area infrared meibography with the Antares topographer. The MG morphology was classified according to the DREAM protocol definitions^10^ (Fig. 2A–D).

The eyelid margin characteristics were classified incrementally, according to Arita et al., as normal, irregular, telangiectasias, orifice obstruction, and displacement of the mucocutaneous junction^31^.

The plugging of the MG opening and MG lid secretions were evaluated by applying mild pressure during 15 s above the five central MG openings of the lower eyelid. Plugging was categorized with the following scoring system: 0 (zero), if all glands were expressible, (1) if 3–4 glands were expressible, (2) if 1–2 glands were expressible, and (3) if no glands could be expressed^8^. In addition, the MGYLS score for the complete eyelid was determined according to Korb et al.^21,32^ Lid secretions expressed from the opening of these glands after application of pressure with a home-made device were classified according to Bron et al. with the following score: (0) clear, (1) opaque, (2) opaque with detritus, and (3) toothpaste-like^33^. The highest grade found among the expressed glands was recorded.

**Figure 3:**
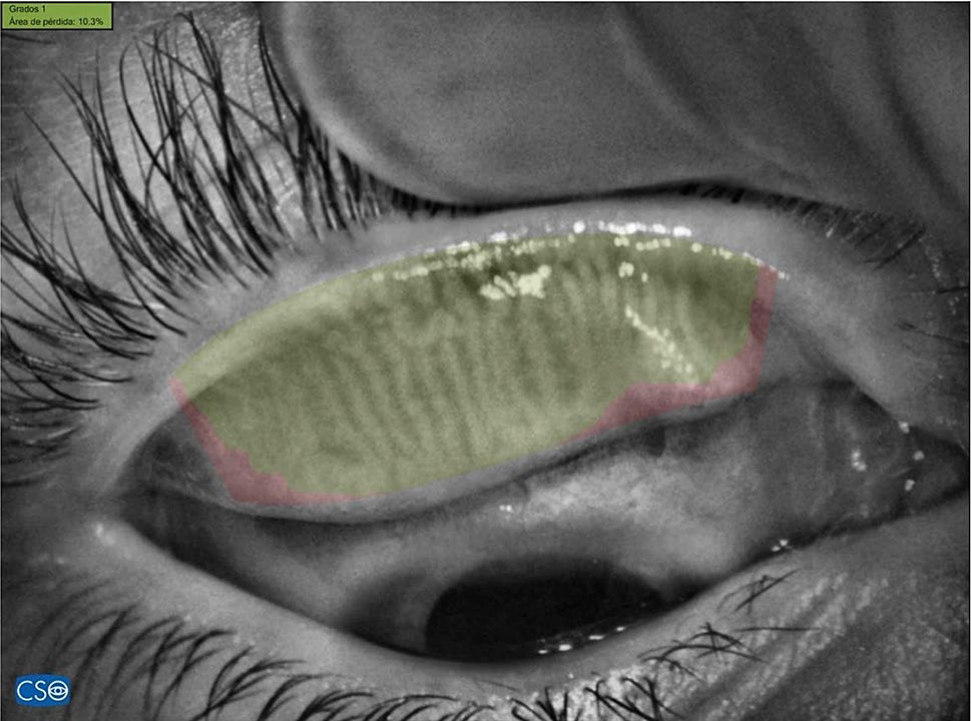
Quantification the percentage of MG loss with the Phoenix software (version 3.2, Construzionne Strumenti Oftalmici, Firenze, Italy), green area: eyelid area with meibomian glands, red area: area with lost of Meibomian glands.

Using the infrared meibography, the morphological characteristics of the MG were observed as hyper-reflective, grape-like acini clusters that are directed toward the palpebral margin in a straight or slightly tortuous line^34^. The area of gland dropout was defined according to Pult et al. by “(1) the actual ending of glands, (2) the width of the area, defined to be between at least from the tear punctum, and the temporal border defined to be to the most well visible tarsal conjunctiva of the everted lid, and (3) the maximal depth of the area was estimated to be where glands would have ended in normal MG morphology,”^21,35^ and also including ghost and fluffy areas, as suggested by Daniel et al.^10^ To quantify the percentage of MG loss, we used the Phoenix software (version 3.2, Construzionne Strumenti Oftalmici, Firenze, Italy) as described in previous reports^34,36^ (Fig. 3).

Two readers graded each lid meibography image independently. The readers were masked to demographic, clinical, and treatment information. Only morphological characteristics that were confirmed by both observers in the same image were included in the analysis. To avoid correlation bias between the two eyes of the same subject^37^, only one eye per subject was included in the analysis. Based on the quality of the meibography image, only images with good or fair quality of lid eversion were used, according with Daniel et al.^10^ If both eyes had the same quality, the most affected eye was included.

### Statistical analysis

Descriptive statistics were obtained for the clinical signs. Paired t-tests and Wilcoxon matched-pairs tests were used to compare data with normal and non-normal distributions, respectively. Upper and lower lid analyses were performed separately. Associations between continuous measures of MG features and signs were evaluated with linear regression, where the MG feature was the dependent variable. Associations between binary measures of MG features were evaluated with logistic regression, where the MG feature was the dependent variable. All regression models involving signs measured on a continuous scale and symptoms were calculated using the continuous values as independent variables. Regression models were adjusted for age and sex. All statistical analyses were performed with SPSS software (v24 for Mac; IBM, Chicago, IL).

We evaluated the correlations between all clinical parameters and morphological characteristics with logistic regression using the RStudio software (v4.0.2 for Windows, RStudio, Boston, MA). We controlled for sex (coded 1 = female; 0 = male) and age to account for factors that might bias our results.

## References

[1] Craig JP, Nichols KK, Akpek EK (2017). TFOS DEWS II definition and classification report. Ocul. Surf..

[2] Stapleton F, Alves M, Bunya VY (2017). TFOS DEWS II epidemiology report. Ocul. Surf..

[3] Garza-León M, Hernández-Quintela E, Cámara-Castillo HG (2017). Prevalencia de síntomas de enfermedad de la superficie ocular en pacientes que acuden a consulta oftalmológica. Gac. Med. Mex..

[4] Sullivan DA, Dana R, Sullivan RM (2018). Meibomian gland dysfunction in primary and secondary Sjögren syndrome. Ophthalmic Res..

[5] Wang Y, Qin Q, Liu B (2019). Clinical analysis: Aqueous-deficient and Meibomian Gland dysfunction in patients with primary Sjogren's syndrome. Front. Med. (Lausanne).

[6] Zang S, Cui Y, Cui Y, Fei W (2018). Meibomian gland dropout in Sjögren's syndrome and non-Sjögren's dry eye patients. Eye (Lond).

[7] Amano S (2018). Meibomian gland dysfunction: Recent progress worldwide and in Japan. Invest. Ophthalmol. Vis. Sci..

[8] Tomlinson A, Bron AJ, Korb DR (2011). The international workshop on meibomian gland dysfunction: Report of the diagnosis subcommittee. Invest. Ophthalmol. Vis. Sci..

[9] Villani E, Marelli L, Dellavalle A (2020). Latest evidences on meibomian gland dysfunction diagnosis and management. Ocul. Surf..

[10] Daniel E, Maguire MG, Pistilli M (2019). Grading and baseline characteristics of meibomian glands in meibography images and their clinical associations in the Dry Eye Assessment and Management (DREAM) study. Ocul. Surf..

[11] Adil MY, Xiao J, Olafsson J (2019). Meibomian gland morphology is a sensitive early indicator of meibomian gland dysfunction. Am. J. Ophthalmol..

[12] Zhao Y, Chen S, Wang S (2018). The significance of meibomian gland changes in asymptomatic children. Ocul. Surf..

[13] Arita R, Itoh K, Inoue K, Amano S (2008). Noncontact infrared meibography to document age-related changes of the meibomian glands in a normal population. Ophthalmology.

[14] Xiao J, Adil MY, Chen X (2020). Functional and morphological evaluation of meibomian glands in the assessment of meibomian gland dysfunction subtype and severity. Am. J. Ophthalmol..

[15] Lee KW, Kim JY, Chin HS (2017). Assessment of the tear meniscus by strip meniscometry and keratograph in patients with dry eye disease according to the presence of meibomian gland dysfunction. Cornea.

[16] Hykin PG, Bron AJ (1992). Age-related morphological changes in lid margin and meibomian gland anatomy. Cornea.

[17] Gu T, Du B, Bi H (2020). Meibomian gland dropout, not distortion, can distinguish dry eyes from normal eyes in contact lens wearers. Curr. Eye Res..

[18] Yin Y, Gong L (2015). Uneven meibomian gland dropout over the tarsal plate and its correlation with meibomian gland dysfunction. Cornea.

[19] Arita R, Fukuoka S, Morishige N (2017). New insights into the morphology and function of meibomian glands. Exp. Eye Res..

[20] Daniel E, Pistilli M, Ying GS (2020). Association of meibomian gland morphology with symptoms and signs of dry eye disease in the Dry Eye Assessment and Management (DREAM) study. Ocul. Surf..

[21] Pult H, Riede-Pult BH, Nichols JJ (2012). Relation between upper and lower lids' meibomian gland morphology, tear film, and dry eye. Optom Vis. Sci..

[22] Eom Y, Choi KE, Kang SY (2014). Comparison of meibomian gland loss and expressed meibum grade between the upper and lower eyelids in patients with obstructive meibomian gland dysfunction. Cornea.

[23] Bilkhu, P., Vidal-Rohr, M., Trave-Huarte, S. & Wolffsohn, J. S. Effect of meibomian gland morphology on functionality with applied treatment. *Cont. Lens Anterior Eye.* 101402 10.1016/j.clae.2020.12.065 (2021). 10.1016/j.clae.2020.12.065 33397598

[24] Singh, S., Naidu, G. C., Vemuganti, G. & Basu, S. Morphological variants of meibomian glands: correlation of meibography features with histopathology findings. *Br. J. Ophthalmol.*10.1136/bjophthalmol-2021-318876 (2021). 10.1136/bjophthalmol-2021-318876 34417185

[25] Mathers WD, Shields WJ, Sachdev MS (1991). Meibomian gland dysfunction in chronic blepharitis. Cornea.

[26] Lin X, Fu Y, Li L (2020). A novel quantitative index of meibomian gland dysfunction, the meibomian gland tortuosity. Transl. Vis. Sci. Technol..

[27] Kim JS, Wang MTM, Craig JP (2019). Exploring the Asian ethnic predisposition to dry eye disease in a pediatric population. Ocul. Surf..

[28] Beltran F, Ramos Betancourt N, Martinez J (2013). Transcultural Validation of Ocular Surface Disease Index (OSDI) Questionnaire for Mexican Population. Investig. Ophthalmol. Vis. Sci..

[29] Peterson RC, Wolffsohn JS, Fowler CW (2006). Optimization of anterior eye fluorescein viewing. Am. J. Ophthalmol..

[30] Whitcher JP, Shiboski CH, Shiboski SC (2010). A simplified quantitative method for assessing keratoconjunctivitis sicca from the Sjögren's Syndrome International Registry. Am. J. Ophthalmol..

[31] Arita R, Itoh K, Inoue K (2009). Contact lens wear is associated with decrease of meibomian glands. Ophthalmology.

[32] Korb DR, Blackie CA (2008). Meibomian gland diagnostic expressibility: correlation with dry eye symptoms and gland location. Cornea.

[33] Bron AJ, Benjamin L, Snibson GR (1991). Meibomian gland disease Classification and grading of lid changes. Eye (Lond).

[34] Garza-Leon M, Ramos-Betancourt N, de la Vega FB-D, Hernández-Quintela E (2017). Meibografía Nueva tecnología para la evaluación de las glándulas de Meibomio. Revista Mexicana de Oftalmología.

[35] Pult H, Riede-Pult B (2013). Comparison of subjective grading and objective assessment in meibography. Cont. Lens Anterior Eye.

[36] Garza-Leon M, Gonzalez-Dibildox A, Ramos-Betancourt N, Hernandez-Quintela E (2020). Comparison of meibomian gland loss area measurements between two computer programs and intra-inter-observer agreement. Int. Ophthalmol..

[37] Murdoch IE, Morris SS, Cousens SN (1998). People and eyes: Statistical approaches in ophthalmology. Br. J. Ophthalmol..

